# Regulatory Uncertainty Around New Breeding Techniques

**DOI:** 10.3389/fpls.2018.01291

**Published:** 2018-09-04

**Authors:** Rim Lassoued, Stuart J. Smyth, Peter W. B. Phillips, Hayley Hesseln

**Affiliations:** ^1^Department of Agricultural and Resource Economics, University of Saskatchewan, Saskatoon, SK, Canada; ^2^Johnson-Shoyama Graduate School of Public Policy, University of Saskatchewan, Saskatoon, SK, Canada

**Keywords:** innovation, uncertainty, gene editing, agricultural biotechnology, European Union, United States, new breeding techniques, food security

## Abstract

Emerging precision breeding techniques have great potential to develop new crop varieties with specific traits that can contribute to ensuring future food security in a time of increasing climate change pressures, such as disease, insects and drought. These techniques offer options for crop trait development in both private and public sector breeding programs. Yet, the success of new breeding techniques is not guaranteed at the scientific level alone: political influences and social acceptance significantly contribute to how crops will perform in the market. Using survey data, we report results from an international panel of experts regarding the institutional and social barriers that might impede the development of new plant technologies. Survey results clearly indicate that regulatory issues, social, and environmental concerns are critical to the success of precision breeding. The cross-regional analysis shows heterogeneity between Europeans and North Americans, particularly regarding political attitudes and social perceptions of targeted breeding techniques.

## Introduction

Modern crop biotechnology has been dynamically progressing through increases in the knowledge about, and applications of, genomics. Scientific advancements have yielded more sophisticated and targeted breeding techniques—known as new breeding techniques (NBTs)—resulting in plants with novel traits including pest and disease resistance, stress tolerance, and improved quality attributes (Sprink et al., 2016). In addition to their simplicity, many NBTs allow clear-cut and reliable mutations, setting them apart from previous genetically modified (GM) crops. The ability to improve crop varieties through the precise addition of useful traits or deletion of undesirable phenotypes (known as gene editing) has to the potential to lower technology development costs and reduce development time (Abdallah et al., 2015). Regardless of their scientific potential, NBTs have been, and are being viewed as a radically controversial innovation in some countries. While some jurisdictions have decided to treat some new plant technologies as simply a variation of existing conventional plant breeding and apply case-by-case assessment (e.g., United States, Canada, Argentina, Brazil, Chile, Columbia, China, Sweden and Australia), others remain mired in uncertainty, unable to determine what to do or how to proceed to regulate (e.g., the EU and France, which both are seeking to use the technology as a trigger).

Regional differences in public expectations and consumer attitudes toward the use of biotechnology in agriculture and its impact on food production and international trade have a lengthy history of examination between the United States and Europe (Gaskell et al., 1999; Jasanoff, 2015; Lau, 2015). Many studies have shown that Europeans’ acceptance of agricultural biotech products is low compared to Americans (Einsele, 2007; Aerni, 2014). As a result, production and consumption policies for transgenic products in the European Union (EU) and North America diverge (Smyth et al., 2013). While the EU endorses the precautionary principle and explicitly incorporates speculative discussion of uncertain risks in its review of GM crops, Canada and the United States focus on managing largely foreseeable risks (Wiener and Rogers, 2002). Why do the EU, Canada and the United States regulate the same technology differently despite their similar economic circumstances as high-income, food exporting nations? In part, the answer lies in public perception (i.e., the subjective assessment of risks and benefits). While Americans have a generally positive attitude on the safety and benefits of biotech crops, most Europeans have a negative opinion (Einsele, 2007). Thus, technology adoption for crop improvement will depend not only on the best scientific method and evidence, but also on effectively and appropriately engaging with the public and industry in the regulatory space (Chapotin and Wolt, 2007).

The innovation literature has largely covered technological and commercial uncertainties, but only superficially explored social debates (Hall et al., 2011). Genetic technology in agriculture has disrupted long-standing acceptance and motivated a range of third parties and stakeholders to engage in the debate. This paper reviews the socio-economic uncertainty triggered by the introduction of NBTs and assesses how this uncertainty influences regulatory assessment and social acceptance of emerging technologies in the agri-food context. Rather than exploring societal concerns from a public or a consumer perspective, we are interested in the cross-cultural differences in expert opinion and, more fundamentally, to what extent do country of origin or field of expertise influence opinions on innovation. We surveyed scientists in industry, government and universities, as well as social scientists. We test whether expert opinions on novel plant biotechnology are influenced by a respondent’s home county as well as to their area of expertise (natural science vs. social sciences).

Using contingency analysis of survey data, this paper deepens the understanding of innovation-related uncertainties of the set of precision breeding tools that are expected to make a crucial contribution to the future of global food security. This paper has five parts: the next section provides a brief theoretical background on innovation and uncertainty; the third elaborates on the research methodology; the fourth presents and discusses the survey results; and this is followed by conclusions.

## Innovation, Regulation, and Uncertainty

Uncertainty is an intrinsic characteristic of innovation as the potential benefits of any specific innovative product or process might be achieved in the future (Jalonen, 2012). In fact, innovations can introduce a wide-range of unintended, often undesirable, health, environmental and social side effects. Risk assessment is a standard approach used to reduce innovation-related uncertainty (Peters et al., 2007). These requirements—with their costs and delays—do not necessarily increase public confidence in biotechnology. Extensive regulatory assessment of plant technologies subject to precautionary principles has led to relatively negative public attitudes to transgenic products (Einsele, 2007; Marchant and Stevens, 2015). Thus, more regulatory oversight might increase public skepticism toward agricultural biotechnology rather than build trust.

The success of agricultural and food innovations depends very much on acceptance by consumers, regulators, and non-governmental organizations (NGOs). The involvement of these secondary stakeholders with conflicting interests creates ambiguity and more complexity (Hall and Martin, 2005). As posited by Aldrich and Fiol (1994), the acceptance of innovation depends on its level of socio-political legitimacy, where cultural aspects and political influences matter. “An innovation thus establishes its legitimacy when its technical performance and social acceptance co-evolves and expands, thus reducing uncertainty” (Hall et al., 2011: 1149). Based on these insights, we emphasize that the legal environment and the social context can either enhance or hinder the success of precision breeding. That is, the success of NBTs is not guaranteed at the scientific level alone, but that political socio-cultural influences significantly contribute to how it will perform in the market.

In the context of plant breeding, in the last two decades scientific progress has created a range of new tools that fall between genetic engineering and conventional techniques (Sprink et al., 2016). Yet, application of NBTs (with its subset of gene editing) lacks legal clarity. One reason could be the large spectrum of NBTs under evaluation. Some techniques are a refinement of conventional breeding, and do not alter the genetic material such as the case of RNA-dependent DNA methylation (RdDM) (HLG-SAM, 2017). Some forms of gene-editing tools including clustered regularly interspaced short palindromic repeats (CRISPR), transcription activator-like effector nuclease (TALEN) and ***zinc-finger nucleases*** (ZFN) induce site-specific genome changes via the development of site-directed nucleases (SDNs). As these point mutations are precision alterations (SDN1 and SDN2), final products are transgene-free and might escape the GM rules (Araki and Ishii, 2015). Other gene editing tools involve gene insertions and are likely to yield transgenic products (SDN3). With the advent of various NBTs and their heterogeneity (e.g., different molecular processes, variety of derived products), countries differ in how they regulate the technologies (Lassoued et al., 2018).

Absence of institutional arrangements governing these new techniques will likely have detrimental effects for their development. In spite of the fact that many European researchers have been leading the development of new crop biotechnology (Eriksson et al., 2018), EU regulatory quandaries around agricultural biotechnology have harshly affected innovation by discouraging scientists from using novel techniques, rejecting research funding applications, and shifting research investment out of the EU (Sprink et al., 2016). In essence, new crops and new technologies cannot prosper without legal authorization. Legal uncertainty creates commercial uncertainty; the more ambiguous are the regulations surrounding NBTs, the more developers are uncertain. Thus, we focus on the regulatory and social uncertainties next.

Regulations and institutional constraints are typically used to protect public health as well as the environment. They may be developed to constrain or support innovation—limiting specific applications or uses through licensing or promoting commercialization through intellectual property rights. The diffusion of novel breeding approaches to crop-trait development depends crucially on appropriate governance of new technologies. Currently, the rules governing agricultural biotechnology do not necessarily directly apply to NBTs; as already noted, that is a policy decision, with different countries making different judgements. As many NBT derived products share phenotypic similarity with conventionally-bred counterparts, logic follows that they should not be classified as regulated forms of GM. Experts have judged that the potential risks of using techniques like gene editing are comparable to conventional and transgenic technologies (EFSA, 2012). And, therein lies the uncertainty around the legal status of NBTs. Except for Canada, most nations tend to assess novel plants based on the process employed rather than the product’s new phenotype, which would likely exempt gene-edited varieties from extensive review. This is an ongoing process in Europe. The 2001 directive governing the release of GMOs in the environment is under interpretation by the European Court of Justice; there is some indication there might be a softening of gene-edited rules, especially when a technique such as CRISPR involves targeted changes to the genome (Abbott, 2018). In the meantime, decisions are made on a case-by-case basis in other parts of the world. In the United States, for instance, authorities exempted many gene-edited crops from GM regulations by providing guidance to product developers through responses to formal review letters (Jones, 2015; Wolt et al., 2016; USDA, 2018). In contrast, the EU has not provided any legal guidance yet for NBT applications (Eriksson et al., 2018). While many NBTs will fall outside the GM regulatory criteria, this may vary by region. We would expect that given the diverging regulatory processes in the United States and EU, that American and European experts might have different opinions on NBTs and their uses.

## Social Uncertainty

Social uncertainty is caused by incomplete information and “is located in the social field, where hesitancy, vagueness, ambiguity or lack of confidence is [are] reflexive characteristics of social objects or actors in a community” (Pillania, 2011, p. 1159). Social uncertainty related to technology refers to whether an innovative product aligns with public values, beliefs and interests. In a way, it is also a judgment of the perception of the performance as well as the competence of social institutions.

A gap exists between the wide-spread farming of biotech crops across the world and the low public acceptance (Lucht, 2015). Despite the historical record on the safety of GM products, consumer opinions around the world are mixed, and social acceptance of biotech products has been limited in many countries. In part, this is due to the reality that the media is the prime source of information available to many consumers. The focus on technological risks in the media, and the vested interests of political stakeholders holding extreme positions, has worked to stigmatize biotechnology in many markets (Aerni, 2002). In addition, European NGOs have been successful in framing biotechnology as a menace. Einsele (2007) argues that the negative reports in newspapers by anti-GM lobbies turned the public against plant biotechnology in Europe.

It is fair to note that global consumer perception of biotech products has been slowly becoming more favorable, especially for output trait (second-generation) GM products that offer consumer health benefits. Earlier studies found that (American) consumers supported transgenic products if they satisfied specific needs such as enhanced nutrition (Hossain et al., 2003), and that they were willing to pay premiums to buy them (Lusk et al., 2003; Kaneko and Chern, 2005). Recent studies have shown that consumers are willing to accept biotech products if transparent information of product safety is shared (Evans and Ballen, 2014). In the same vein, some assert consumers will welcome products of NBTs if labeling adheres to the “Right to Know” rule.

Agricultural biotechnologies such as gene editing might be viewed differently in different countries, resulting in different regulatory and market decisions. It is expected that the highest degree of uncertainty lies within the social dimension (which is arguably the most complex) as there are more groups to accommodate (e.g., local, national and international communities, environmental activists). In addition to a consumer’s mindset, social uncertainty is affected when civil society movements question the safety or efficacy of novel technologies (Paarlberg, 2014). One example of this was in 2015, when the European Academies’ Science Advisory Council (2015) advised EU regulators that NBT-derived products, which are free of foreign gene(s), do not require GM regulation. Anti-GMO NGOs called on the Commission to ensure that NBTs be regulated within the current GM legislation framework (NGO-coalition, 2015). Thus, adoption of precision breeding could be hampered by public understanding and social acceptance rather than by technological aspects (Araki and Ishii, 2015).

Awareness of and appreciation for the benefits of these viable alternatives to transgenic crop breeding methods for crop improvement might reduce regulatory oversight (Wolt et al., 2016). If novel plant traits are not understood and accepted by the public, political pressure to have them evaluated under GM biosafety rules will increase, decreasing the availability of NBTs to public breeders in many, if not most, countries.

## Materials and Methods

The data used for the analysis reported in this paper stems from two online surveys examining the socio-regulatory aspects of uncertainty as it relates to NBTs. The regulatory survey was emailed to an expert panel of 638 on January 2016, and the social survey was emailed to 630 participants in May 2017. Both surveys have run for a 4-month period each with biweekly reminders. The questionnaires have comparable structures, asking respondents to rank the limiting factors to the development of NBTs, and to identify their sources of confidence used to form opinions.

These surveys are part of a multi-year project investigating risk preferences among experts regarding innovative plant breeding^1^. The target population includes scientists, regulators, and business professionals with backgrounds and experiences in agricultural biotechnology. A contact database was constructed using emails of participants in from a number of conferences on GM technology organized by the researchers dating over the past 15 years, and of experts from online searches (university websites, biotechnology research institutions, governmental agencies websites, etc.). Recruiting a large panel of international experts online is a challenging task: this method allowed us to reach out to a large number of international experts in the field of study.

In October 2015, an introductory recruitment effort was conducted. Those that enrolled in the research panel provided socio-demographic information and answers to a series of decision-making questions (survey materials are available on the website). Prospective panelists were asked about their primary current job and to identify themselves as scientist, regulator, policy advisor, economist, etc. Based on the answers, the researchers grouped the panelists into scientists—mostly according to plant/natural sciences, and social sciences. An expertise variable was used in the analysis to compare groups of experts. Respondents were also asked about their country of residence (chosen from a drop-down menu). For analytical purposes, the countries were grouped into three regions: North America, Europe and the rest of the world.

Our study (BEH 97) was exempt from full ethics review by the Behavioral Ethics Board at the University of Saskatchewan on April 7, 2015. The exemption status was based on the fact that the participants are not themselves the focus of the research per the Tri-Council Policy Statement: Ethical Conduct for Research Involving Humans, December 2014, Exemption Article 2.1.

## Results and Analysis

This section reports survey results on the sample characteristics and the contingency analysis. The questionnaires on the regulatory and social uncertainties of NBTs were completed by 201 and 173 respondents, yielding response rates of 31.5 and 27.5% respectively. Tabulated statistics and Chi-square analysis are reported on two categorical variables: expertise and region. The variable expertise includes two groups: scientists or scientific experts (about 40% of the sample), and non-scientists and social scientists, including regulators and industry professionals (about 60%). Considering the size of our sample, we aggregated results to regions rather than countries, as the Chi-square statistic is sensitive to sample size (i.e., it needs large expected frequencies). The variable region includes North America (NA: Canada and United States: about 50%), Europe (25%), and the rest of the world (ROW: Asia, Africa, Oceania, Central and South America: 25%). We assess the differences in opinions between groups and regions with respect to NBT-related regulatory and social uncertainties.

The panel is dominated by males (79%), aged between 45 and 65 years (70%). As mentioned above, nearly half of the panelists reside in North America, a quarter in Europe, and the remainder in the ROW (5% from Central and Latin America, 5% from Australia and New Zealand and 3% from Africa). The majority of subjects hold a PhD degree (71%); 20% have a masters’ degree. Eighty percent are employed and 14% are self-employed. Forty percent work for industry, 26% for university, and 20% for government. Panelists were asked about the type of crops and markets they work with. Main crops of interest include cereals (63%), oilseeds (43%), pulses (39%) and vegetables (25%). More than 70% of the sample works with both food and feed, 43% on fiber, 37% on industrial ingredients, and 29% on environmental services.

Below, we report survey results on the regulatory and social uncertainties. We would like to briefly mention that while we did not report the technical uncertainty of NBTs here, we conducted a survey on the topic. Key results show that intellectual property (IP) and patents, public funding and technological uncertainty were deemed the top three major hurdles to the development of most novel techniques. In addition, 60% of participants felt moderately confident answering the questions related to the scientific uncertainty of NBTs. A further 21% felt very confident. About one fifth lacked confidence. Results suggest that respondents have moderate to high confidence in their answers thus reflecting knowledge of new breeding techniques. We report detailed results about the regulatory issues as the highest degree of uncertainty lies within these dimensions.

## Regulatory Uncertainty Results

Participants were asked about the regulation of NBT techniques. As displayed in **Table 1**, over half of the sample (52%) indicated that some crops generated via precision breeding should be regulated as GM products whereas 32% believe they should not be regulated as such. Only 16% consider NBT derived crops to be like, or similar to, transgenic crops. Survey results show that respondents believe products of synthetic biology and of targeted gene editing techniques involving gene insertions or substitutions should fall in the same regulatory space as products produced by transgenesis.

**Table 1:** Opinions of appropriate regulation of NBT derived crops, differentiated by region and type of respondent (% of total).

	*Regions*			Total	*Expert groups*	
	NA	Europe	ROW		Scientists	Non-scientists
NBT derived products should be regulated as GM technology	10	2	4	16	7	9
NBT derived products should not be regulated as GM technology	18	7	6	32	12	20
Some NBT derived products should be regulated as GM technology while others should not	20	16	17	52	23	29
Total	48	25	27	100	42	58
***Chi-square statistic***	χ^2^ = 8.578; *p* = 0.073				χ^2^ = 0.797; *p* = 0.671	

We conducted cross-tabulation for both region and expertise; those with *p*-values greater than 0.05 indicate statistical independence of the variables of interest. There is no statistically significant difference in the opinions about how NBTs should be regulated among the three regions. Indeed, the majority of the sample (52% that specifically includes 20, 16, and 17% of North Americans, Europeans and the ROW, respectively) agrees that some NBT products should be regulated as GM products while others should not. Similarly, expertise is not found to affect responses. Similar proportions of scientists and non-scientists share opinions about the regulation of NBT-derived products. Despite the diverging regulatory systems around the world that govern biotechnology (i.e., process-based system in Europe, product-based system in Canada, and a hybrid system in United States), experts did not differ about how NBT techniques should be regulated. According to Marchant and Stevens (2015), nations should move toward a product-based approach as it would be more sustainable for newer methods of crop breeding.

Respondents were provided with a list of factors that might explain innovation-related regulatory uncertainty. They were invited to rank up to five factors they thought were the most limiting to the development of NBTs. One-quarter of the sample indicated that political involvement in the regulatory process, followed by unsynchronized approval between countries, are the most limiting factors facing NBTs (See **Table 2**). Inconsistent international standards, incomplete national regulatory rules, high regulatory compliance costs, and regulatory delays were other critical factors affecting emergence of NBTs.

**Table 2:** Regulatory barriers to the development of NBTs.

Limiting factors	Percentage
Political involvement in regulatory process	24
Unsynchronized approval between countries	20
Inconsistent international standards	19
Incomplete national regulatory rules	17
High regulatory compliance costs	17
Regulatory delays	16
Lack of skilled staff among regulators	7
Lack of scientific evidence	7
Inadequate infrastructure to carry out experiments and/or field trials	4
Lack of baseline data	6
Shortage of staff among regulators	3
Inadequate funding	3
Overly rigorous confidential business information	3

*The score is a weighted sum value of the 5 ranked responses. Items ranked first were multiplied by 0.5. Ranks 2, 3, 4, and 5 were weighted 0.4, 0.3, 0.2, and 0.1, respectively*.

Participants were asked to rank seven proposed sources of confidence they might rely upon to form their answers about the regulatory uncertainty of NBTs. The survey revealed that half of the sample tended to rely on their personal experience (54%), information from regulatory agencies (48%) and from academic studies (42%). It is interesting to note that information from NGOs was mentioned by 24% of respondents (See **Table 3**). When asked how confident they felt in answering the regulatory uncertainty question, 40% were moderately confident and 36% were very confident. Less than a quarter of the sample was slightly confident and only 5% were not confident.

**Table 3:** Trusted sources of information on regulatory matters.

Sources of confidence	Percentage
My personal experience	54
Information from national regulatory agencies	48
Information from academic studies	42
Information from international regulatory agencies	41
Information from advisory bodies	37
Information from companies	37
Information from NGOs	24

*The score is a weighted sum value of the 7 ranked responses where 1st, 2nd, 3rd, 4th, 5tg, 6th, and 7th choices were weighted 0.7, 0.6, 0.5, 0.4, 0.3, 0.2, and 0.1, respectively.*

The panel was asked whether their domestic government would adopt policies in line with their views (**Table 4**). Respondents seem to fall into two main groups—those who think that their government will (*definitely* and *probably*) adopt policies in line with their views (57%), and those who think that their government will (*definitely* and *probably*) not align with their views (43%). The crosstabs show some regional divergence (*p* < 0.001). The majority of NA and ROW respondents, including 31% (representing 60% of NA respondents) and 18% (representing 75% of ROW respondents) respectively, think their governments will adopt policies in line with their views, while the majority of Europeans (16%, which represents 67%) think the opposite. This is not surprising given the rigid nature of EU legislation toward crop biotechnology. There was no evidence that experts diverged with respect to policy adoption: a majority of scientists (23%, which represents 58%) and of non-scientists (34%, which represents 57%) think that their domestic government will (*definitely* and *probably*) adopt policies in line with their views.

**Table 4:** Policy alignment between expert view and government regulation, by region and group (% of total).

	*Regions*			Total	*Expert groups*	
	NA	Europe	ROW		Scientists	Non-scientists
*(Probably/Definitely)* Yes	31	8	18	57	23	34
*(Probably/Definitely)* No	21	16	6	43	17	26
Total	52	24	24	100	40	60
***Chi-square statistic***	χ^2^ = 15.278, ***p* < 0.001**				χ^2^ = 0.042, *p* = 0.837	

*The scale options “Probably yes” and “Definitely yes” were recoded as “Yes” to increase the cell count. Same for “No.” The recoding does not affect the result interpretation. Bold value indicates significant p-values at 0.05.*

When asked about the likelihood of approving NBTs, 52% indicated that they are either optimistic or very optimistic, while 15% were pessimistic or very pessimistic. Almost a third were neutral in their views. Contingency analysis in **Table 5** shows that respondents exhibited different levels of optimism regarding the likelihood of approving NBTs depending on their home region. The majority of North Americans (29%, which represents 56%) were optimistic, while Europeans (17%, which represents 74%) were more pessimistic or neutral. The current strict EU legal regime governing agricultural biotechnology—mainly based on the precautionary principle—might contribute to this divergence. There is no evidence that experts diverge, as the majority of both groups of experts (52%) are optimistic about the likelihood of approving NBTs in the future.

**Table 5:** Opinions on likelihood of governments approving NBTs, differentiate by region and group (% of total).

	*Regions*			Total	*Expert groups*	
	NA	Europe	ROW		Scientists	Non-scientists
Optimistic/Very optimistic	29	6	17	52	23	29
Neutral	18	8	7	33	11	22
Pessimistic/Very pessimistic	5	9	1	15	6	9
Total	52	23	25	100	40	60
***Chi-square statistic***	χ^2^ = 31.392, ***p* < 0.001**				χ^2^ = 1.793, *p* = 0.408	

*To increase the cell count, the scale options “Very optimistic” and “Optimistic” were grouped together. Similarly for “Very Pessimistic” and “Pessimistic”. Bold value indicates significant p-values at 0.05.*

## Social Uncertainty Results

Participants were asked to rank a list of socially-related factors that could limit the success of precision breeding. About one-third of the sample (34%) ranked public perceptions—led by social objections—as the most critical obstacle to the development of NBTs, followed by food/human safety concerns (mainly toxicity and allergenicity) at 27%, and environmental concerns (e.g., increased use of chemicals in agriculture and loss of biodiversity) at 21%. Animal/feed safety concerns were identified as a limiting factor by only 12%.

Panelists were asked about the five most important sources of confidence they used to form their answers. Results of **Table 6** show that university scientists are the most highly trusted at 29%. Regulators (18%), farmers/farmer organizations (17%) and environmental groups (16%) were closely grouped. Retailers (2%), private firms (3%), ethics committees (3%) and medical doctors (4%) ranked quite low. This finding confirms the significance of scientific evidence on the subject of innovative breeding.

**Table 6:** Trusted sources of information and judgment on social aspects of NBTs.

Sources of confidence	Percentage
University scientists	29
Regulators	18
Farmers/Farmer organizations	17
Environmental groups	16
Industry associations	12
Consumers’ organizations	12
Advocacy groups	12
Social media websites	8
Politicians	5
Medical doctors	4
Other	4
Firms	3
Ethics committees	3
Religious leaders	2
Retailers	2
Primary education system	2

As shown in **Tables 7** and **8**, 70% of the experts think that people from their country perceive some benefits from products obtained via precision breeding, against 90% who think that people perceive some risks from these products. In **Table 9**, 54% of the respondents indicated that people believe that NBTs can (*definitely/probably*) improve global food security. Contingency analysis shows that Europeans do not agree with other countries about the perceived benefits of NBT products (*p* < 0.001); moreover, they do not believe NBTs have much potential to address food insecurity (*p* = 0.006). Specifically, 45% of non-European respondents, but only 9% of their European counterparts, said that people in their countries (*Probably/Definitely*) believe NBTs could improve global food security. These findings demonstrate a great uncertainty on the future of precision breeding in Europe. The regional heterogeneity in opinions about NBTs is likely to affect the regulatory process as well as the global trade of crop commodities. Non-scientists believe people in general perceive almost no risks related to NBTs while 7% of scientists believe people do perceive some risks (*p* = 0.002).

**Table 7:** Opinions of fellow citizens regarding perceived benefits from NBT derived products among regions and among experts (% of total).

	*Regions*			Total	*Expert groups*	
	NA	Europe	ROW		Scientists	Non-scientists
(Probably/Definitely) Yes	41	12	16	70	25	45
(Probably/Definitely) No	8	15	7	30	15	15
Total	49	27	23	100	40	60
***Chi-square statistic***	χ^2^ = 18.069, ***p* < 0.001**				χ^2^ = 2.592, *p* = 0.107	

*Bold value indicates significant p-values at 0.05.*

**Table 8:** Opinions of fellow citizens regarding perceived risks from NBT derived products among regions and among experts (% of total).

	*Regions*			Total	*Expert groups*	
	NA	Europe	ROW		Scientists	Non-scientists
(Probably/Definitely) Yes	47	25	19	90	33	58
(Probably/Definitely) No	2	3	4	10	7	2
Total	49	28	23	100	40	60
***Chi-square statistic***	χ^2^ = 5.542, *p* = 0.063				χ^2^ = 9.364, ***p* = 0.002**	

*Bold value indicates significant p-values at 0.05.*

**Table 9:** Opinions of fellow citizens regarding perceived food security among regions and among experts (% of total).

	*Regions*			Total	*Expert groups*	
	NA	Europe	ROW		Scientists	Non-scientists
(Probably/Definitely) Yes	32	9	14	54	17	38
(Probably/Definitely) No	17	18	10	46	23	22
Total	49	27	24	100	40	60
***Chi-square statistic***	χ^2^ = 10.241, ***p* = 0.006**				χ^2^ = 9.364, ***p* = 0.016**	

*Bold value indicates significant p-values at 0.05.*

**Table 10** shows that more than half of the panelists agree that communicating the benefits and risks of NBTs to the public should be a shared responsibility among university scientists (85%), regulators (75%), farmers/farmer organizations (64%), consumer organizations (53%), and industry associations (52%). These responsible institutions were also the most trusted sources experts use to form their opinions on precision breeding. This refers to the congruity principle (Osgood and Tannenbaum, 1955) by which “we tend to trust institutions who share our attitudes” (Peters et al., 2007: 196).

**Table 10:** Responsible institutions for sharing the benefits and risks of NBTs.

Institutions	Percentage
University scientists	85
Regulators	75
Farmers/Farmer organizations	64
Consumers’ organizations	53
Industry associations	52
Environmental groups	47
Politicians	36
Primary education system	35
Ethics committees	31
Advocacy groups	26
Social media websites	25
Medical doctors	24
Firms	21
Retailers	17
Religious leaders	7

When asked about the likelihood that people would willingly purchase NBT-derived products, over half of the respondents think that it is (e*xtremely/moderately*) likely that consumers in their country will buy such products when available on the market; 10% think it is unlikely. While the crosstabs of **Table 11** indicate no difference in opinions by background, there is some evidence of different views by region. NA and the ROW show higher likelihood of consumers purchasing NBT products. In the United States, and since the introduction of GM crops, many consumers were little to not concerned about biotech products and were willing to buy GM products despite their superficial knowledge regarding plant biotechnology (IFIC, 2006). Unlike NA, European respondents appear to be less positive about future purchases of NBT products. In fact, the majority (17% that represents 61%) is either neutral or thinks it is unlikely that consumers will choose such products. On the other hand, 40% of Europeans are likely to try NBT products. This suggests that not all Europeans exhibit resistance to biotech products obtained via modern plant breeding. This is in line with existing research showing that not all Europeans are suspicious about biotech products. For instance, Aerni et al. (2011) found that Swiss consumers purchased GM corn bread when having the opportunity to choose freely between GM and non-GM variants.

**Table 11:** Likelihood of consumers buying NBT products, by region and group (% of total).

	*Regions*			Total	*Expert groups*	
	NA	Europe	ROW		Scientists	Non-scientists
(Extremely/Moderately) Likely	41	11	16	68	25	43
Neither likely nor unlikely	8	9	5	22	8	14
(Extremely/Moderately) Unlikely	1	8	1	10	6	4
Total	50	28	22	100	39	61
***Chi-square statistic***	χ^2^ = 29.661, ***p* < 0.05**				χ^2^ = 4.163, *p* = 0.125	

*Bold value indicates significant p-values at 0.05.*

In summary, we found more statistical differences based on region than on expertise. The groups of experts (natural science vs. social science) disagree on the perceived risks posed by NBTs and their potential to address global food insecurity. Non-scientists hold attitudes that are more positive. Unsurprisingly, findings show that the European respondents have the perception that the EU is socially and politically more precautionary about the application of new plant gene technology compared to the rest of the world; other studies of public attitudes and regulatory decisions tend align with that view. Europe seems to be less positive about the likelihood of approving, and adopting, NBTs. In addition, expert opinions in the EU indicate that consumers are less likely to purchase NBT-derived products due to the lack of perceived benefits.

## Conclusion

Scientific innovation in the world of biology, particularly new techniques for breeding plants, are advancing rapidly. The ability to move from random mutation through the application of chemical or radiation mutation breeding to the precision of point-specific mutation offered through new breeding techniques is challenging regulatory systems to respond in a timely manner. The results presented and discussed above offer insights into the challenges of resolving this regulatory gap.

The regulatory uncertainty pertaining to products of NBTs is not due to scientific concerns, but rather political interference in the regulatory approval process. As identified above, the top reasons for uncertainty regarding regulatory approval of varieties produced by innovative plant breeding have no connection to science. The first scientific concern identified in the list of uncertainties was ranked by only 7% of respondents. The experts are clearly indicating that if the regulation of gene-edited technologies was to occur strictly based on scientific risk assessment principles, that these products would safely receive approval. But with political interference in the regulatory approval process, most notably in the EU, many express concerns that there will be few successful approvals.

Experts in the EU are less confident than are experts in other parts of the world, most notably North America, that consumers will accept NBT products. Some of our results support the fact that the EU is often described as being inflexible to the adoption of gene technology, including transgenic crops. Yet, we recognize there is variation among the EU countries regarding both political and public attitudes to plant gene technology. About 8–10 countries (of the EU-28) tend to be highly restrictive while 8–10 (e.g., Scandinavian and northern European) have a more pragmatic, science-based approach to GM applications (see Eriksson et al., 2018). These differences in opinions are not grounded in science, but rather in politics.

The results of our expert surveys reveal that trust in science is strong, while trust in social structures lags considerably. Our expert panel is not confident that politicians will not interfere in the regulatory approval for the products of new breeding technologies, thus increasing the uncertainty regarding the successful use of the technology. Given the highly competitive market for strategic agricultural and food investments, the level of uncertainty that exists within the EU has the potential to divert potential research and development investment away from the EU to markets with greater regulatory certainty.

## Data Availability

The raw data supporting the conclusions of this manuscript are not publicly available because academic survey policy at the University of Saskatchewan states that all personal survey data will be protected and held confidential to ensure responder anonymity. Requests to access these datasets should be directed to Dr. Stuart Smyth at stuart.smyth@usask.ca.

## Author Contributions

RL developed the method, performed the analysis, and wrote the paper. SS helped developing the method, writing the paper, and supervised the study. PP and HH helped developing the method and writing the paper.

## Conflict of Interest Statement

The authors declare that the research was conducted in the absence of any commercial or financial relationships that could be construed as a potential conflict of interest.

## Footnotes

1 https://research-groups.usask.ca/nbt-regulation/
